# On the Morbid Effects of the Loss of Blood

**Published:** 1830-07-01

**Authors:** 


### VIII.

**RESEARCHES PRINCIPALLY RELATIVE TO THE MORBID AND CURATIVE EFFECTS OF LOSS OF BLOOD. By *Marshall Hall*, M.D. 8vo. 1830.**

ONE object of Dr. Hall's researches is to shew the remarkable difference in the degree of tolerance or intolerance of loss of blood in different diseases—another object, and a very important one, is to establish the distinction between inflammations and irritations. Some observations on these subjects were published by our author ten years ago; but he has now enlarged on these topics, and added the testimonies of several living practitioners, in support of his conclusions.

The morbid effects of blood-letting have certainly not been studied so well as the curative, though they are not less interesting—syncope, delirium, convulsions, coma, and subsequent excessive reaction, are among the most conspicuous features of these bad effects of bleeding.

1. *Syncope.* It would appear that the primary cause of this phenomenon is defect of circulation in the brain, since posture alone relieves it; but then the deficiency of power in the heart must precede the affection of the brain. The respiration, the state of the stomach and bowels become secondarily affected. The efforts to vomit are succeeded by an amelioration of the syncope. The reaction which next takes place, may be excessive or defective—or it may be wanting altogether, and yield to an opposite condition—*sinking*.

2. *Convulsion.* Next, after syncope, the most familiar of the immediate effects of blood-letting, is convulsion. It is more apt to occur in children, and in cases of slow and excessive detraction of blood. This is evidently dependent on affection of the brain—it is a phenomenon which shews that the brain may be "similarly affected by opposite states of the general system" —an observation as old as Hippocrates. It is evident that the constitutional treatment must be diametrically opposite in the two cases of repletion and exhaustion. Dr. H. conceives that convulsion occurring after blood-letting, usually denotes that too much has been taken away, often in the recumbent position, when a great deal of blood flows before deliquium is induced.

*Case.* "A physician, aged thirty-four, became affected with inflammation of the larynx. He was bled freely on two successive mornings at his own instance. In the afternoon of the second day, the disease being unsubdued, he was bled a third time, placed in a rather inclined position upon a sofa. The blood was allowed to flow until thirty-four ounces were taken. He then suddenly fell upon the floor violently convulsed; and he remained for some time afterwards in such a state of syncope as to render his recovery very doubtful; being carried to bed, however, and cordials being administered, he slowly recovered. He did not afterwards suffer from the secondary effects of loss of blood.

"A similar case is given by Mr. Travers. This gentleman observes, 'Some patients cannot bear the loss of blood ; it gives rise to prostration, attended with convulsions, in which the circulation fails so alarmingly as to require watching for several hours and the repeated administration of stimulants to restore it. A very intelligent surgeon in the neighbourhood of London, in bleeding a clergyman to the extent of twenty ounces, whose idiosyncracy in this respect was not known, was compelled to remain with him during the whole of that day ; and notwithstanding frequent recourse to brandy, continued long apprehensive for the patient's life. He represented the convulsions, which returned in paroxysms, as resembling the puerperal in their severest form. There has been reason to believe that the loss of blood in operations in which hæmorrhage was unavoidable, has sometimes induced this state ; this however, in the present advanced state of surgery is rare.' " 16.

3. *Delirium.* This occurs as an immediate effect of loss of blood, whereas mania is a more remote effect. Dr. Hall mentions the case of a young man who had lost much blood from the arm and by leeches, besides being briskly purged. He fell into complete syncope. Instead of laying him recumbent, they kept him erect, and thus protracted the deliquium for an hour and a half. He was found colourless and senseless, affected with rattling in the breathing. On being laid down the blood began to come to his face, and animation to increase. The pulse was quick, and he complained of deafness. To these phenomena succeeded severe rigor, great heat of skin, and delirium. These symptoms gradually subsided, and the patient recovered. Several other cases of an analogous nature are adduced as exemplifications of the injurious effects of inordinate loss of blood.

"Mrs. ——, miscarried on the third month of her pregnancy. There was considerable flooding. On unthinkingly getting out of bed for some purpose, there was a sudden profuse gush of blood. She turned pale and nearly fainted. She was promptly carried and laid upon the bed, but soon became affected with convulsion. This was succeeded by delirium, which continued during two hours. A little brandy and water was given. She recovered in a few days.

"As might have been anticipated, delirium frequently occurs as an immediate effect of hæmorrhagy during parturition. Still more frequently mania occurs as a remote effect of loss of blood.

"It is important to remark that delirium may occur even from the loss of a very small quantity of blood, in those cases in which there is what I have ventured to term intolerance of loss of blood, or in other words, great susceptibility to its effects, a subject which will be fully discussed in the second part of this work.

"Mrs. ——, aged 40, had been, for some time, under medical treatment for a small tumour in the mamma. She was seized with rubeola ; on the first and second days of the rash she was purged freely, too freely ; on the third she was bled to eight ounces, and six leeches were applied to the chest, for a slight oppression felt there. Mrs. ——, had also passed five nights totally without sleep, although on the evening of the second day she had taken twenty-five minims of the tinctura opii.

"Under the influence of these circumstances, Mrs. ——, was seized with delirium. I saw her on the fourth day ; there were constant delirium, a profuse perspiration, a trifling rash, and a feeble pulse of 120. I gave fifty drops of tinctura opii, and one dram of the spiritus ammoniæ aromaticus.

"I saw Mrs. ——, again in four hours ; the delirium had subsided into a state of obstinate silence, the patient sitting up in bed, refusing to answer questions, and having had no sleep ; the skin and pulse as before. I directed one ounce of brandy to be given every hour, with beef tea.

"The first dose of brandy had produced sleep. It was directed to be continued every hour, at my visit in the morning.

"On the morning of the second day I was informed that eight ounces of brandy had been taken during the night; and that there had been much quiet sleep. I found the patient collected, the pulse 108, and less feeble; the skin still in a state of perspiration. The brandy was directed to be given every second hour. In the evening there was still further amendment. The bowels not having been moved, an aperient draught was prescribed to be taken early in the morning.

"This draught acted thrice. The delirium returned, and was removed by the brandy, which again procured sleep.

"From this time there was no recurrence of delirium. The perspiration kept profuse for some days, but gradually subsided; the pulse became gradually slower and stronger. There was afterwards a protracted affection of the chest." 22.

4. *Coma.* We are sometimes called to patients who are perfectly comatose after bleeding, and we are in doubt whether the case be or be not apoplexy. The history, the countenance, the pulse, the state of the extremities, and other symptoms, will help to guide us, after a little careful reflexion.

5. *Sudden Dissolution.* For examples of this the reader is referred to Dr. Hall's Commentaries on the Diseases of Females.

In the second chapter, the author treats of the more remote effects of loss of blood, or exhaustion—and first of exhaustion with excessive reaction.

"The reaction or recovery from ordinary syncope is generally a simple return to a healthy state of the functions, or nearly so, the pulse not passing beyond its natural frequency. In cases of profuse loss of blood, on the contrary, the recovery is not quite so uniform, and the pulse acquires and retains a morbid frequency for a certain length of time; this frequency of the pulse may gradually subside, however, and be unattended by any other symptom of indisposition of any consequence.

"The phenomena are very different, if, instead of one full bleeding to syncope, or of a profuse hæmorrhage, and even protracted syncope, the person be subjected to repeated blood-lettings or to a continued drain. In this case, within certain limits, the pulse, instead of being slow and feeble, acquires a morbid frequency and a throbbing beat, and there are, in some instances, all the symptoms of excessive reaction.

"This state of excessive reaction is formed gradually, and consists, at first, in forcible beating of the pulse, of the carotids, and of the heart, accompanied by a sense of throbbing in the head, of palpitation of the heart, and eventually perhaps of beating or throbbing in the scrobiculus cordis, and in the course of the aorta. This state of reaction is augmented occasionally by a turbulent dream, mental agitation, or bodily exertion. At other times it is modified by a temporary faintness or syncope. There is also sometimes irregularity of the beat of the heart and of the pulse.

"In the more exquisite cases of excessive reaction the symptoms are still more strongly marked, and demand a fuller description.

"The beating of the temples is at length accompanied by a throbbing pain of the head, and the energies and sensibilities of the brain are morbidly augmented; sometimes there is intolerance of light, but still more frequently intolerance of noise and of disturbance of any kind, requiring stillness to be strictly enjoined, the knockers to be tied, and straw to be strewed along the pavement; the sleep is agitated and disturbed by fearful dreams, and the patient is liable to awake or to be awoke in a state of great hurry of mind, sometimes almost approaching to delirium ; sometimes there is slight delirium, and occasionally even continued delirium ; more frequently there are great noises in the head as of singing, of crackers, of a storm, or of a cataract ; in some instances there are flashes of light ; sometimes there is a sense of great pressure or tightness in one part or round the head, as if the skull were pressed by an iron nail or bound by an iron hoop.

"The action of the heart and arteries is morbidly increased, and there are great palpitation, and visible throbbings of the carotids, and sometimes even of the abdominal aorta, augmented to a still greater degree, by every cause of hurry of mind or exertion of the body, by sudden noises or hurried dreams or wakings ; the patient is often greatly alarmed and impressed with the feeling of approaching dissolution ; the state of palpitation and throbbing are apt to be changed, at different times, to a feeling of syncope ; the effect of sleep is in some instances very extraordinary, sometimes palpitation, at other times a degree of syncope, or an overwhelming feeling of dissolution ; the pulse varies from 100 to 120 or 130, and is attended with a forcible jerk or bounding of the artery." 31.

The respiration is apt to be hurried, and attended with alternate panting and sighing—" the movement of expiration being sometimes obviously blended with a movement communicated by the beat of the heart." The skin is sometimes hot, and hurry and restlessness prevail. In this state of exhaustion, sudden dissolution is sometimes the immediate consequence of any muscular exertion, or even of being raised from the horizontal posture. The following case exemplifies some of the effects of loss of blood in a striking manner.

"Mrs. ——, aged 28, of a stout constitution. After delivery there was uterine hæmorrhagy, which continued to recur for the twelve subsequent months. It was then discovered that Mrs. ——, laboured under polypus uteri ; a ligature was applied, purgative medicines given, and the patient recovered. The effects of this loss of blood followed, however, and there were, 1. beating of the temples, a sense of violent 'knocking' in the head, pain, vertigo, dimness of sight, and singing in the ears, terrific dreams, and starting from sleep ; 2. frequency of the pulse, pulsation of the carotids and aorta, fluttering and beating of the heart, faintishness, and a sense and fear of dissolution ;—the palpitation of the heart was sometimes such on awaking as even to move the bed clothes, the bed, and, it is said, even the door ; 3. the breathing was short and hurried, sometimes with panting, sometimes with sighing ; 4. there were urgent calls for air, for opened windows, and the smelling bottle, and the nostrils and temples were required to be bathed with sal volatile or vinegar.

"The countenance, prolabia, and tongue were pallid ; the legs somewhat œdematous ; the bowels were irregular, the secretions morbid ; once there was obstinate constipation ; frequently the bowels were merely confined, sometimes with sickness, but always with an increase of all the symptoms." 33.

Exhaustion with defective reaction is the next subject of consideration. The opposite phenomena are mostly observed in young people of robust constitution, who have been subjected to repeated blood-letting. In infants, feeble persons, and persons of advanced age, reaction, after loss of blood, is apt to be defective. In this case the patient remains long pale, thin, and feeble, becoming faint on the slightest occasions. The pulse is frequent, but feeble, and perhaps irregular ; and we have not the throbbing and palpitation so common in the opposite circumstances.

### EXHAUSTION WITH SINKING.

"The symptoms of exhaustion with excessive reaction may gradually subside and leave the patient feeble but with returning health; or they may yield to the state of sinking. This term is adopted not to express a state of negative weakness merely, which may continue long and issue in eventual recovery, but to denote a state of positive and progressive failure of the vital powers, attended by its peculiar effects, and by a set of phenomena very different from those of exhaustion with reaction.

"If in the latter the energies of the system were augmented, in the former the functions of the brain, the lungs, and the heart are singularly impaired. The sensibilities of the brain subside, and the patient is no longer affected by noises as before; there is, on the contrary, a tendency to dozing, and gradually some of those effects on the muscular system which denote a diminished sensibility of the brain supervene, as snoring, stertor, blowing up of the cheeks in breathing, &c.; instead of the hurry and alarm on awaking as observed in the case of excessive reaction, the patient in the state of sinking, requires a moment to recollect himself and recover his consciousness, is perhaps affected with slight delirium, and he is apt to forget the circumstances of his situation and, inattentive to the objects around him, to fall again into a state of dozing.

"Not less remarkable is the effect of the state of exhaustion with sinking on the function of the lungs; indeed the very first indication of this state is, I believe, to be found in the supervention of a crepitus in the respiration, only to be heard at first on the most attentive listening; this crepitus gradually becomes more audible and passes into slight rattling, heard in the situation of the bronchia and trachea; there is also a degree of labour or oppression, sighing, hurry, blowing, in the breathing, inducing acuteness in the nostrils, which are dilated below and drawn in above the lobes at each inspiration; in some cases there is besides, a peculiar catching, laryngeal cough, which is especially apt to come on during sleep, and awakes or imperfectly awakes the patient.

"The heart has, at the same time, lost its violent beat and palpitation, and the pulse and arteries their bounding or throbbing.

"The stomach and bowels become disordered and flatulent, and tympanitic, and the command over the sphincters is impaired.

"The last stage of sinking is denoted by a pale and sunk countenance, inquietude, jactitation, delirium, and coldness of the extremities." 46.

### EXHAUSTION WITH DELIRIUM.

This section contains only a case communicated to the author by Dr. Abercrombie.

We conceive that it was neither more nor less than a case of puerperal mania, and had little or nothing to do with the leechings which had been employed for the topical affection. As the case is short, we shall here give it.

"'A lady aged about 35, after delivery of her fifth or sixth child, recovered favourably for a fortnight, when she became affected with pain and deep-seated hardness in the right side of the pelvis, painful to the touch, and accompanied by a considerable degree of fever. After repeated topical bleeding, and various other remedies, the febrile state subsided, the swelling lost its tenderness, and it seemed to be gradually diminishing in size; but its progress was slow; and, after three or four weeks from the time of its appearance, she was still confined to bed, and was suffering a good deal of uneasiness: her pulse was now calm, but she was considerably reduced in strength. At this time she was one day alarmed and agitated by some family occurrence of a trifling nature, and immediately began to talk wildly and incoherently; and, after a restless night, she was found next day in the highest state of excitement, talking incessantly, screaming, and struggling, with a wild expression of countenance, and a small rapid pulse. She was now treated by leeches to the temples, cold applications to the head, purgatives, &c. but with little or no benefit; and, on visiting her next day, I found her sitting up in bed, with a look of extreme wildness, both her hands in constant motion; she was talking incessantly, loudly, and wildly, and I learnt that she had not ceased talking in this manner for one instant for the last twelve hours. Her pulse was rapid and feeble, and her countenance expressive of exhaustion. This was a state which I had repeatedly seen fatal under various modes of treatment, and I certainly expected no good of the case. But in consultation with a highly respectable medical friend who had charge of it, I proposed to try a treatment purely stimulant. A full glass of white wine was accordingly given, and ordered to be repeated every hour. On visiting her at the end of the fourth hour, I found her composed and rational; her pulse 90 and of good strength; and from this time there was no return of the symptoms. The tumour in the right side increased in size, suppurated, was opened, and healed favourably; and she is now in perfect health." 60.

### Exhaustion, with Coma.

The following case was communicated to Dr. Hall by a friend. An opulent farmer, 60 years of age, tall and well formed, of active habits in the early part of life, and of sanguineous temperament, had suffered severely from gout, and for many years had suffered from spasms about the cardiac region, attended with urgent sickness and giddiness—in fact, "gout in the stomach." The biliary system was much deranged and jaundice had occurred. He had more than once been attacked with pneumonia, requiring one or more general bleedings. About five months before the fatal event, he suffered an apoplectic seizure, and his life was saved by active depletion. From that time his health became unsettled and his whole frame enfeebled. Depression of spirits was extreme, and he was harrassed with cramps and sub-acute gout.

"After exposure in an open carriage, on one fine day, with the wind in the east, he was suddenly attacked with a severe pain in the abdomen, chiefly in the right hypochondrium and towards the stomach, the respiration being at the same time exceedingly painful and laboured. His medical attendant viewed the disease as acute hepatitis, and bled him profusely from the arm, and with immediate relief, which was rendered more complete by the free action of purgative medicines. There was a slight recurrence of the symptoms in the course of forty-eight hours, and large depletion from the vessels was again practised, so that at the end of three days, nearly seventy ounces of blood had been abstracted." 67.

This active work, in an old and enfeebled man, soon depressed the vital powers. The patient appeared bloodless and cadaverously pallid. The tongue was pale, the pupils dilated, the sight imperfect, the eyes glassy. There was subsultus tendinum, jactitation of the limbs, suspirious respiration, pulse 100 to 120, irritable and throbbing. Cordials were exhibited, the powers of life revived, and the patient recovered so far as to be able to take airings in his carriage. But complete convalescence was never re-established, and in the course of a month he died suddenly. No examination of the body was obtained.

Amaurosis sometimes becomes the prominent symptom in exhaustion from loss of blood, of which Mr. Travers gives some good examples in his Synopsis of Diseases of the Eye. The following is an interesting case.

"A young medical man came to me one morning from the country in extreme anxiety, with an earnest solicitation that I would instantly apply a ligature to his carotid artery. This gentleman aged 25, was of short stature, and constitutionally healthy. His pupils were large and his countenance was suffused, and bore the appearance of preternatural determination of blood to the head. He had been the subject of two attacks of inflammation; one in April, the other in October of the same year; during which he lost upwards of an hundred ounces of blood. He had now a constant heavy pain in the head chiefly over the coronal suture, and in the direction of the sinuses, with tinnitus of the left ear. After stooping the giddiness was extreme, and a golden-coloured spot, edged with black, appeared floating before the eye. He had been troubled with muscæ in excess, for a year and a half past; he had now fire sparks flashing before the sight, and saw a pulse in the choroid synchronous with that of the wrist. When looking at near objects he was not troubled with muscæ, but they were always numerous, in proportion as the object was remote. He did not complain of much dimness. His complaints were not relieved by topical blood-letting. He recovered gradually but perfectly, under a regulated diet, and a course of the blue pill with saline aperients." 71.

The amaurosis from depletion is sometimes mistaken for its opposite—plethoric congestion. This is owing to the coincidence of a dilated pupil—muscæ volitantes, a deep-seated pain in the head, with occasional vertigo. It succeeds, according to Mr. Travers, somewhat abruptly to uterine floodings, and large depletion for acute diseases. The pain is not confined to the region of the orbit, though it generally affects but one side of the head.

"It is that peculiar nervous pain to which women are subject after uterine hæmorrhage, attended with a sense of defined pressure, as of an iron finger on the brain; and sometimes a distressing jarring noise like that of a mill or threshing-floor, or the rattling of the shingles as a heavy wave of the sea recedes. It is perhaps connected with an imperfect injection of the medullary substance. By a cautious use of tonics it is relieved; by whatever lowers or stimulates, whether diet or medicine, it is decidedly aggravated. The vision in this form of amaurosis is further enfeebled by the loss of as much blood as flows from two or three leech-bites. This is not imaginary; I have seen distinctly marked cases of it, in which large and copious venesection was still urged as the only resource of art. This I consider to be a fatal mistake." 72.

The fourth chapter is on "the *further* loss of blood in cases of exhaustion." Dr. H. is convinced, and so are we, that the symptoms of exhaustion, with re-action, are frequently mistaken for inflammation or other disease of the head or the heart. Under this impression, further recourse is had to the lancet—and, as the symptoms are perhaps greatly relieved, the wrong view of the case is confirmed.

"It was some time before I could fully comprehend the nature of this fact. I had satisfied myself that, in certain cases, the symptoms were those of loss of blood; and yet it appeared no less certain that those very symptoms were relieved by the lancet. At length I discovered, by a careful observation, that the symptoms which were relieved were those of re-action; and that the mode of relief was by the substitution of syncope; that the relief endured as long as the state of faintishness continued, but returned as this state gave way to the rallying and re-action of the vital powers.

"Another circumstance equally interesting and curious was, that within certain limits, the remedy which relieved for a time, eventually only added to the severity of the malady, for this was apt to return after a certain period, in a still more aggravated form. It is natural, indeed, to suppose, that, unless there was a tendency to the failure of the vital powers, the re-action of the system and the painful circumstances attending it, would be greater after a third or fourth loss of blood, than after a first or second; indeed there are seldom the symptoms of re-action after one flow of blood, however great or profuse; it is the repetition or protraction of the cause which, as I have already observed, is essential to produce this effect.

"It is observable too that in cases of exhaustion with re-action, syncope is very soon produced by the further loss of blood. This fact is of importance, because it may be regarded as a sign of the state of exhaustion, when this is obscured by the re-action of the system, and as a warning voice against the further and inconsiderate use of the lancet." 75.

We must pass on to the 7th chapter, on the treatment of the various effects of loss of blood. Syncope, re-action, and sinking, each require the appropriate treatment.

"The constitutional treatment must be stimulant in syncope, sedative and soothing in the state of re-action, and restorative in that of sinking. The local treatment must vary with the organ chiefly affected, and with the mode in which it is affected.

"When syncope assumes a dangerous form, the principal remedies are, an attention to the posture of the patient, stimulants, and chiefly brandy, and the transfusion of blood.

"The effect of posture is not, even now, fully known. It would be easy to allow the patient to lie over the edge of the bed, the head low upon the floor, and the feet greatly raised. In this manner such pressure would be restored to the encephalon as would in many cases support life, until, other remedies being administered, the patient might be placed out of immediate danger.

"I need not, in this place, notice the importance of a regulated mode of giving brandy and nourishment. I think it is frequently given in such quantities as actually to induce sickness, and its own rejection from the stomach, and so as to frustrate the object of the physician completely. The effect should be carefully watched. The physician ought not, of course, in such a case, to leave the patient for a moment.

"The next remedy is transfusion. Unfortunately it has too frequently happened that the proper period of adopting this measure has been allowed to pass by. Not only the vascular system is exhausted, but, after a time, the functions of the nervous system have begun to fail. It might be a question, therefore, whether galvanism might not be usefully conjoined with transfusion." 190.

In case of excessive re-action, extreme quiet of body and mind is necessary—then the mildest sedatives, especially hyosciamus—lastly, gentle nutriment and TIME.

The second part of the work, on the "curative effects of loss of blood," we must reserve for another article.

